# Axonal myelin decrease in the splenium in major depressive disorder

**DOI:** 10.1007/s00406-018-0904-4

**Published:** 2018-07-06

**Authors:** Matthew R. Williams, P. Sharma, C. Macdonald, R. K. B. Pearce, S. R. Hirsch, M. Maier

**Affiliations:** 1 Robert Steiner Unit, Hammersmith Hospital, London, W12 0NN UK; 2 Neuropathology Unit, Division of Experimental Medicine, Imperial College London, Charing Cross Campus, St Dunstan’s Road, London, W6 8RP UK; 3 Ophthalmology Department, East and North Hertfordshire NHS Trust, Lister Hospital, Coreys Mill Lane, Stevenage, SG1 4AB UK; 4 KHPC Biobank, Innovation Hub, Guy’s Cancer Centre, Great Maze Pond London, SE1 9RT UK; 5 Claybrook Centre, West London Mental Health NHS Trust, Claybrook Road London, W6 8LN UK; 6 Trust HQ, West London Mental Health NHS Trust, Uxbridge Road Southall, UB1 3EU UK

**Keywords:** Depression, Neuropathology, Axons, Splenium, Myelin

## Abstract

The corpus callosum has become a key area of interest for researchers in severe mental illness. Disruptions in fractional anisotropy in the callosum have been reported in schizophrenia and major depressive disorder. No change has been reported in oligodendrocyte density and overall size of the callosum in either illness, suggesting that gross morphology is unchanged, but subtler organisational disruption may exist within this structure. Using high-resolution oil immersion microscopy, we examined the cross-sectional area of the nerve fibre and the axonal myelin sheath; and using standard high-resolution light microscopy, we measured the density of myelinated axons. These measurements were made in the splenium of the corpus callosum. Measures were taken in the sagittal plane in the callosal splenium to contrast with the previous similar examination of the callosal genu. Cases of major depressive disorder had significantly decreased mean myelin cross-sectional area (*p* = 0.014) per axon in the splenium than in controls or schizophrenia groups. There was no significant change in the density of myelinated axons. The results suggest a clear decrease of myelin in the axons of the callosal splenium in MDD, although this type of neuropathological study is unable to clarify whether this is caused by changes during life or has a developmental origin. In contrast with increased myelin in the callosal genu, this result suggests a longitudinal change in callosal myelination in major depressive disorder not present in normal or schizophrenic brains.

## Introduction

The corpus callosum (CC) is the largest white matter (WM) structure in the CNS, running along the midline of the brain and uniting the two cerebral hemispheres. The thickest and most posterior portion of the CC is termed the splenium of the corpus callosum (spCC), which curves ventrally and continues anteriorly. It overlaps with the tela choroidea of the third ventricle and the posterior part of the dorsal midbrain. Axonal fibres comprising the CC join the cerebral hemispheres by way of homotopic and heterotopic connections, with a majority of commissural fibres uniting symmetrical areas of the hemispheres, although a proportion terminate in different contralateral regions from which they emanate. The spCC primarily connects regions of the parietal and occipital lobes [1], although the anterior part contains projections from medial temporal association areas [2].

The splenium itself is roughly composed of two primary fibre groups, composed of primarily reciprocal fibres from the temporal association and parietal association areas [2–5], although animal research has suggested that splenium fibres play a role regulating thalamocortical connections to the visual cortex [5, 6].

A significant callosal thinning was restricted to the genu in the early onset patients, but patients with late-onset depression exhibited a significant callosal thinning in both genu and splenium relative to controls. The splenium of the corpus callosum was also significantly thinner in subjects with late- vs early onset depression. Genu and splenium midsagittal areas significantly correlated with memory and attention functioning among late-onset depressed patients, but not early onset depressed patients or controls. Circumscribed structural alterations in callosal morphology may distinguish late- from early onset depression in the elderly [7].

The splenium of the CC connects the posterior cortices with fibres varying in sizes from thin late-myelinating axons in the anterior part, predominantly connecting parietal and temporal areas, to thick early‘myelinating fibres in the posterior part, linking primary and secondary visual areas. Splenium function in the human brain defined by the specialization of the area and implemented via excitation and/or suppression of the contralateral homotopic and heterotopic areas at the same level or different level of visual hierarchy. These mechanisms are facilitated by interhemispheric synchronization of oscillatory activity, also supported by the splenium. In the early childhood, myelination of the splenium correlates with the formation of interhemispheric excitatory influences in the extrastriate areas, whilst increasing inhibitory effects in the striate cortex are linked to the local inhibitory circuitry. Reshaping interactions between interhemispherically distributed networks are thought to lead to a reduction of metabolic and structural redundancy in the human brain [8].

Anatomical abnormalities in the CC in depression have been reported in diffusion weighted imaging [9, 10] and magnetisation transfer imaging [11–13], as MRI signal intensity can be used as a putative index of corpus callosum myelination. Bipolar patients had lower corpus callosum signal intensity for all callosal subregions (genu, anterior and posterior body, isthmus, splenium) than controls, whilst a whole brain myelin decrease has been reported in MDD, focused particularly in the dlPFC and NAcc [14]. Both SZ and MDD have been demonstrated to have changes in the CC. In vivo diffusion tensor imaging (DTI) studies have shown decreased fractional anisotropy (FA) in and across the WM in SZ [15], specifically in the spCC but not the callosal genu (gCC) [16–18]. First episode SZ has been reported to not be changed in callosal FA [19], but with decreased size of the gCC, body, and spCC, in contrast to other findings of overall increased thickness in chronic SZ [20–22]. DTI studies have shown that treatment-resistant MDD has lowered callosal FA, which is not related to illness severity [23, 24], and that FA was significantly decreased in the gCC in MDD, as well as bipolar disorder [25, 26]. Neuropathological studies of the callosum have shown no change in axon density, oligodendrocyte density, or overall gliosis in MDD or SZ, although, previously, we and others have reported an increase in axonal myelin in the callosal genu (gCC) in MDD [27–30].

Myelin-related and oligodendroglial genes exhibit decreased transcriptional activity in post-mortem tissue studies, suggesting oligodendroglial dysfunction in SZ. Microarrays have shown a significant reduction in expression of myelination-related genes MAG, PLLP, PLP1, gelsolin, and ERBB3 in SZ [31]. MAG regulates myelination and the development of myelin-producing cells in the CNS [32], whilst ERBB3 encodes neuregulin receptor Her3 that is required for oligodendrocyte survival and differentiation, and gelsolin is involved in stabilising the actin cytoskeleton of axons [33]. The hippocampus and cingulate cortex, both strongly related to the structure and function of the fornix and gCC, caudate, putamen, and multiple cortical regions also show decreased expression of myelin-related genes and oligodendrocyte markers in SZ [34, 35].

Oligodendrocyte-gene expression is not altered in MDD in thalamic or striatal regions, but two astrocyte markers, GFAP and ALDH1L1, are increased across thalamic and striatal regions in both SZ and MDD. In contrast microarray examination of temporal lobe, samples from MDD show 17 genes with altered expression, 16 of which are involved in myelin formation of oligodendrocyte regulation [32, 36]. Meta-analyses has shown decreased expression of MAG, ERBB, TF, PLP1, MOBP, and MOG across multiple brain regions, reviewed in Sokolov [37].

Protein studies suggest that many signalling proteins, such as ephrin B and ciliary neurotrophic factor, and those associated with cell growth and maintenance such as neurofilaments and tubulins, cell communication and signalling, and oligodendrocyte function such as myelin basic protein and myelin-oligodendrocyte glycoprotein are differentially phosphorylated and expressed in the CC in SZ [38, 39]. These results overlap with other post-mortem studies on proteins in SZ and suggest alternative significance of these changes in SZ as well as MDD.

This study aims to examine the myelin thickness, axonal internal size, and density of myelinated axons by means of novel high-resolution light microscopy using an optimised histological stain in the spCC in well-characterised cohorts of SZ, MDD, and controls using precisely optimised histological staining and high-resolution oil immersion microscopy.

## Methods

### Cases

Sagittal blocks containing the spCC were obtained from the Corsellis Tissue Bank in West London Mental Health Trust UK, according to strict inclusion/exclusion criteria. All the brains come from the Corsellis Brain Collection, consisting of over 6500 brains collected during the period 1952–1997. The collection consists of brains of individuals whose death was reported to the coroner’s court reported by Professor JAN Corsellis and retained for further research. The majority of samples come from the county of Essex, but a smaller number came from national referrals [40]. Medical notes were reviewed by a consultant psychiatrist and patients were selected on fulfillment of the WHO International Classification of Disease 10th edition (ICD-10) criteria, for SZ and MDD. For robustness of diagnosis of the brains of the three diagnostic groups, all suffered from chronic psychiatric illness or no recorded incidence of such in the case of controls. Assessment for any neurodegenerative, neurovascular, or infectious pathology, including Parkinson’s disease, was undertaken by a consultant pathologist and affected patients excluded. Patients with any recorded history of alcohol or drug abuse were also excluded. In SZ selection, the presence of first-rank symptoms was a necessity, and cases with onset older than 30 were excluded. Age- and post-mortem interval (PMI)-matched controls were selected as far as possible within the selection criteria. 59 cases were examined overall. 20 controls, 23 SZ, and 16 MDD, were included in the study (individual details in Table 1 and group means in Table 2).

**Table 1:** Summary of clinical and histological information on the brains from the Corsellis collection

Case no.	Sex	Age	PMI	TiF/year	Diagnosis	Cause of death
1	F	36	36	27	NPD	Acute myocardial infarction
2	M	38	29	24	NPD	Chronic ischemic heart disease
3	M	38	NK	29	NPD	NK
4	M	41	NK	27	NPD	Lymphoid leukemia
5	F	45	23	18	NPD	Diffuse non-Hodgkin’s lymphoma
6	M	51	NK	25	NPD	Hodgkin’s disease
7	F	52	NK	24	NPD	Pneumonia, organism unspecified
8	F	52	NK	23	NPD	Malignant neoplasm of stomach
9	F	55	NK	29	NPD	Other diseases of digestive system
10	F	56	NK	27	NPD	Malignant neoplasm of breast
11	F	56	NK	29	NPD	Chronic renal failure
12	F	56	NK	28	NPD	Malignant neoplasm of bronchus/lung
13	F	58	98	14	NPD	Chronic ischemic heart disease
14	F	58	30	26	NPD	Acute myocardial infarction
15	F	59	NK	27	NPD	Gastric ulcer
16	F	59	NK	25	NPD	NK
17	F	60	NK	18	NPD	NK
18	F	60	NK	26	NPD	Acute pericarditis
19	F	60	NK	28	NPD	Myeloid leukemia
20	F	60	NK	28	NPD	Pneumonia, organism unspecified
21	M	47	29	35	SZ	Acute myocardial infarction
22	M	48	46	14	SZ	Intentional self-harm by smoke/fire
23	F	49	26	35	SZ	Acute myocardial infarction
24	M	50	80	14	SZ	Pneumonia
25	F	50	40	15	SZ	Malignant neoplasm of bronchus/lung
26	M	52	16	22	SZ	Pneumonia, organism unspecified
27	M	54	19	29	SZ	Pneumonia, organism unspecified
28	M	54	22	32	SZ	Cardiomyopathy
29	F	54	NK	29	SZ	Drowning
30	M	56	58	35	SZ	NK
31	F	56	126	26	SZ	Congestive heart failure
32	M	56	63	33	SZ	Pneumonia, organism unspecified
33	M	56	NK	31	SZ	Gastric ulcer
34	F	57	23	30	SZ	Acute myocardial infarction
35	F	57	93	17	SZ	Benign CNS neoplasm
36	M	57	8	22	SZ	Heart failure
37	M	59	28	26	SZ	Congestive heart failure
38	F	59	119	38	SZ	Heart failure
39	F	59	19	28	SZ	Intentional self-harm by hanging
40	F	59	41	36	SZ	Chronic renal failure
41	F	59	26	30	SZ	Acute myocardial infarction
42	M	60	24	29	SZ	Acute myocardial infarction
43	M	59	45	27	SZ	Pneumonia, organism unspecified
44	F	41	28	21	MDD	Intentional self-poisoning
45	M	42	26	12	MDD	Asphyxiation
46	F	44	41	12	MDD	Asphyxiation
47	F	45	66	18	MDD	Intentional self-poisoning
48	F	47	NK	20	MDD	Intentional self-harm by hanging
49	M	48	17	18	MDD	Suicide - CO2
50	M	52	73	23	MDD	Intentional self-poisoning
51	M	54	40	22	MDD	Intentional self-harm by hanging
52	F	55	63	15	MDD	Intentional self-poisoning/narcotics
53	F	55	28	34	MDD	Pulmonary embolism
54	M	56	NK	21	MDD	Intentional self-harm by jumping object
55	F	57	NK	25	MDD	Intentional self-poisoning with gases
56	M	57	21	26	MDD	Pneumonia, organism unspecified
57	F	58	62	23	MDD	Acute myocardial infarction
58	M	58	NK	19	MDD	Pulmonary embolism
59	F	60	NK	21	MDD	Pneumonia

Diagnostic codes from ICD-10

*PMI* post-mortem interval, *TIF* time in formalin, *NPD* no psychiatric disorder, *SZ* schizophrenia, *MDD* major depressive disorder, *NK* not known

**Table 2:** Summary group data

Diagnostic Group	Age/year	Sex (M:F)	PMI/h	TiF/year
NPD	52.5 (1.85)	4:16	43.2 (6.93)	25.1 (0.91)
SZ	55.1 (0.82)	13:10	45.3 (6.97)	27.5 (1.49)
MDD	51.8 (1.58)	7:9	42.3 (5.06)	20.6 (1.35)

Age, PMI, and fixation time shown as means with SEM in brackets. The SZ group was significantly older than the MDD group (*p* = 0.012), and in formalin for a longer period (*p* = 0.021)

*PMI* post-mortem interval (*p* = 0.739), *TiF* time in formalin, *NPD* no psychiatric disorder, *SZ* schizophrenia, *MDD* major depressive disorder, *NK* not known

### Dissection and tissue processing

**Fig. 1:**
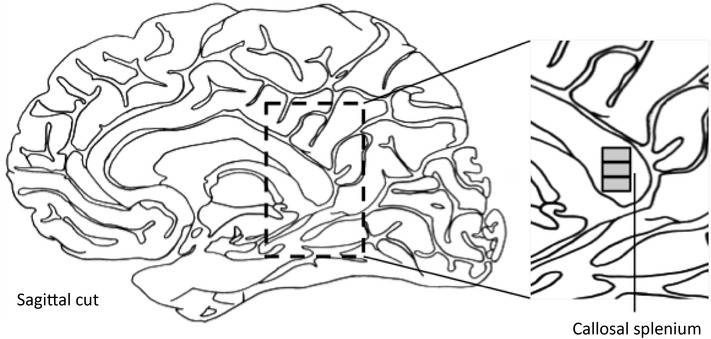
Illustration of neuroanatomy of study. The highlighted region is the callosal splenium from which measures were taken, enlarged below. Grey boxes in sagittal section of genu are the regions of measurement

The spCC was dissected in the sagittal plane from selected cases (Fig. 1) 10% formalin (4% formaldehyde v/v) and stored in 10% formalin (4% formaldehyde v/v), before tissue processing. Blocks were processed using a Leica C300 tissue processor on a routine 12 h programme. Blocks were paraffin-embedded, sectioned at 10 µm thickness, and mounted on 25 × 75 mm electrostatic glass slides. For each case, 3 × 10 µm slides at 100 µm intervals per region were used for staining. Cases were blinded by an independent investigator before measurement.

### Histological staining

One 10 µm section from each region was stained with hematoxylin and eosin (H&E). The sections were incubated in xylene for 15 min, then hydrated in 100, 90, and 70% ethanol (EtOH) and distilled water (dH_2_O) for 2 min each. Sections were immersed in 50% Mayer’s hematoxylin for 5 min before being rinsed in tap water (tH_2_O) and differentiated in 70% EtOH/1% hydrochloric acid (HCl) by immersing the sections for 3 × 1 s. Sections were incubated in 1% eosin for 5 min, rinsed in tH_2_O, dehydrated through serial alcohols, immersed in xylene, and mounted using DPX.

Two 10 µm sections per region were stained with Luxol Fast Blue (LFB). These were de-waxed in xylene and immersed in two washes of 100% EtOH for 2 min each time. Sections were immersed in 1% LFB/methanol solution overnight in a 60 °C oven. Sections were rehydrated in 100, 90, and 70% EtOH and rinsed in tH_2_O until water ran colourless. Sections were differentiated in saturated lithium carbonate solution, dehydrated through serial alcohols, immersed in xylene, and mounted using DPX.

All cases were blinded by an independent investigator before image capture and measurement.

### Image capture

Sections stained with H&E were used for neuroanatomical guidance; adjacent LFB-stained slides were used for axonal measures. All assay and measurement images were captured at 2096 × 1536 resolution using an Olympus Vanox AHBT 3 microscope with a Q Imaging Micropublisher RTV 3.3 camera and analysed using Image-Pro Plus 5.1 software (Media Cybernetics, US). Density measurements were taken at 400× total magnification, whilst axonal architecture measures were taken at 1000× under oil immersion.

### Microscopy

LFB-stained sections from the splenium were used for all measures with H&E sections as an anatomical guide. Images for axon measures were all taken with oil immersion at 1000× magnification. Three images per slide were taken under the criterion that they contain more than three axons, so that enough measures could be obtained. Axons had to exhibit clearly defined myelin rings that were darker than both the inside of the axon and the surroundings, so that they could be traced accurately and without difficulty. A total of eight axons were measured from each region of each LFB-slide, a number determined previously to be the optimum number of neuroarchitectural measures to make for maximum efficiency [29, 41, 42]. Images were taken at 1000× magnification from the exact centre of each region on each of the two LFB-slides per case for density measures (as shown in Fig. 1).

### Measures

Measures were taken according to the results of calibration assays previously described [29]. All visible transverse axons in each image were tagged and then randomly selected using a random number generator. In each of the three sampled regions from each of the slides, regions shown in grey in Fig. 1, a total of randomly selected eight axons were measured for myelin thickness and internal axon diameter as described by the assay protocol.

For each case, there were 20 measurements per axon, 8 axons per region, 3 regions per slide, and 2 slides per case. This gave a total of 960 measures on 48 randomly selected axons per case. All measures of each type were pooled before analysis.

Cross-sectional area (CSA) measures of internal axon diameter and myelin area were measured on static images taken at 1000× as described above. Area was calculated by appropriate calibration the image to the magnification, and by tracing along the inner and outer edges of the sheath the area of the myelin ring. The calibrated computer software gave CSA in µm^2^.

### Data analysis

Slides were unblinded after all measures had been collected and data collected between different slides for the same region and case was merged for analysis. All statistical analyses were performed using Windows SPSS 16.0. The four measures of mean myelin thickness, myelin CSA, mean axon thickness, and axon CSA were examined independently in the spCC. Each of the measures was analysed using univariate analysis with general linear model (GLM).

The primary comparison was changes between diagnostic groups, with diagnosis along with sex and age examined in the GLM as interacting variables. In addition, in each analysis using the GLM potential confounders such as fixation period, incidence of suicide and PMI were examined as confounding variables. As a secondary analysis following significant findings, direct tests between single groups were performed using *t* tests.

### Ethics

This project was conducted under ethical permission granted by the London south west local ethics committee reference WL/02/12 (2002), and amendment WL/02/12/AM01, granted by the Ealing and WLMHT local research ethics committee (2006).

## Results

The SZ group was significantly older than the MDD group (*p* = 0.012), and in formalin for a longer period (*p* = 0.021; see Table 2). There was a significant interaction in GLM analysis between age and myelin CSA (*p* = 0.038), although regression analysis showed no direct relationship between age and myelin CSA in the spCC (*p* = 0.377, *r*^2^ = 0.00052). There was no significant effect of age, sex, PMI, or formalin-fixation period interacting with any other confounding variable or upon myelin thickness, internal axonal diameter, or axon density in the splenium. There was no effect of the incidence of anti-psychotic treatment (*p* = 0.836) or chlorpromazine-equivalent units with presented results (*p* = 0.600), and no effect of anti-depressant treatment (*p* = 0.644).

The incidence of suicide is unevenly distributed between the groups (controls *n* = 0, SZ *n* = 2 confirmed, it was unclear whether drowning in case 29 was accidental, MDD *n* = 9). Within the groups, there was no interaction of suicide on the measurements (SZ, *p* = 0.249; MDD, *p* = 0.603).

**Fig. 2:**
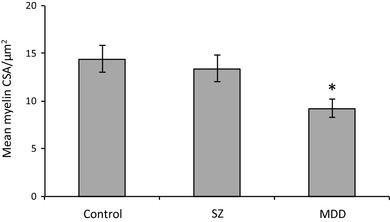
Graph of spCC myelin CSA. *p < 0.05 (control: 14.4 ± 1.4 µm^2^, MDD: 9.2 ± 1.0 µm^2^, 36% decrease, *p* = 0.015). *SZ* schizophrenia, *MDD* major depressive disorder

**Fig. 3:**
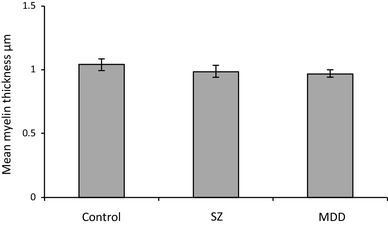
Graph of spCC myelin thickness (control: 1.04 ± 0.05 µm, MDD: 0.97 ± 0.03 µm, *p* = 0.413). *SZ* schizophrenia, *MDD* major depressive disorder

MDD brains showed significantly smaller myelin CSA (control: 14.4 ± 1.4 µm^2^, MDD: 9.2 ± 1.0 µm^2^, *df*_8,46_, 36% decrease, *p* = 0.015; *F*_crit 8,46_ = 2.15; Fig. 2) but not myelin thickness (control: 1.04 ± 0.05 µm, MDD: 0.97 ± 0.03 µm, *df*_8,46,_*p* = 0.413; *F*_crit 8,46_ = 2.15; Fig. 3) in the spCC compared to SZ and control.

**Fig. 4:**
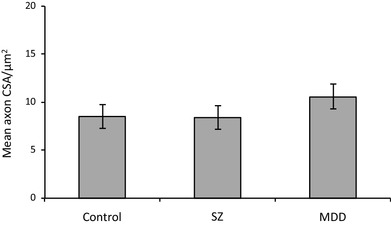
Graph of spCC axon internal CSA (control: 8.5 ± 1.3 µm^2^, MDD: 10.5 ± 1.3 µm^2^, *p* = 0.411). *SZ* schizophrenia, *MDD* major depressive disorder

**Fig. 5:**
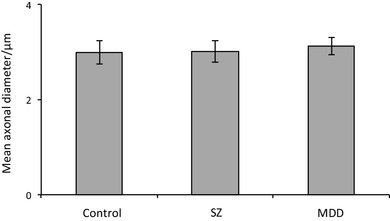
Graph of spCC axon internal diameter (control: 3.0 ± 0.25 µm, MDD: 3.1 ± 0.18 µm, *p* = 0.900). *SZ* schizophrenia, *MDD* major depressive disorder

There was no change in axonal internal CSA (control: 8.5 ± 1.3 µm^2^, MDD: 10.5 ± 1.3 µm^2^, *df*_8,46,_*p* = 411; *F*_crit 8,46_ = 2.15; Fig. 4) or internal axonal diameter in MDD compared to the other diagnostic groups (control: 3.0 ± 0.25 µm^2^, MDD: 3.1 ± 0.18 µm^2^, *df*_8,46_; *p* = 0.900; *F*_crit 8,46_ = 2.15; Fig. 5).

There was no significant change in the density of myelinated axons between diagnostic groups in the spCC (*p* = 0.182).

There was no change in the spCC in SZ between diagnostic groups in myelin CSA (spCC, *p* = 0.672), myelin thickness (spCC, *p* = 0.445), internal axonal CSA (spCC, *p* = 0.273), internal axonal diameter (spCC, *p* = 0.654), or in axon density (spCC, *p* = 0.806).

The results are summarised in Table 3.

**Table 3:** Summary of findings shown as means with SEM in brackets

Diagnostic group	Myelin CSA/µm^2^	Axon internal CSA/µm^2^	Myelin thickness/µm	Axonal diameter/µm
NPD	14.4 (1.4)	8.5 (1.3)	1.04 (0.047)	3.00 (0.25)
SZ	13.4 (1.4)	8.4 (1.2)	0.99 (0.046)	3.00 (0.23)
MDD	9.2 (1.0)*	10.6 (1.3)	0.97 (0.028)	3.12 (0.18)

*p < 0.05 (control: 14.4 ± 1.4 µm^2^, MDD: 9.2 ± 1.0 µm^2^, 36% decrease, *p* = 0.015)

*NPD* no psychiatric disorder, *SZ* schizophrenia, *MDD* major depressive disorder, *NK* not known

## Discussion

The results demonstrate a significant decrease in the myelin CSA of spCC axons in MDD, with no change observed in axon size or density. This change is the opposite effect observed in the gCC, suggesting an anterior–posterior change in axon structures along the callosum in MDD not present in SZ or controls.

### Biological significance

The results presented could indicate in hemispheric connection via the spCC between the parietal or occipital lobes in MDD. The thinner myelin suggests slower neural transmission rates in MDD in the splenium, as healthy myelin coating of nerve axons is related to signal transmission speed, although as axons carry inhibitory signals as well as excitatory ones, this effect may not be clinically obvious. Whilst no structural changes in the parietal or occipital lobes have been reported in schizophrenia [43], an increase in occipital malformation where one lobe bends around the other has been reported in depression [44]. A neurodevelopmental disorder such as this could possibly affect the development of reciprocal connections through the splenium. Low GABA concentrations have also been reported in the occipital lobe in depression, possibly reflecting a functional change in this structure that may affect maintenance of the myelin on associated networks.

Whilst myelin thickness is determined by oligodendrocyte function, there is no direct evidence published to suggest decreased oligodendrocyte activity in this region. Decreases in myelin- and oligodendrocyte-gene expression have been reported in MDD [32, 36, 37], but functional gene network modelling has suggested that this may not indicate large-scale disruption of the gene networks, and, hence, physiological function, as many changes may be to compensate for by other as yet undiscovered alterations [45]. Such reported changes may compensate for one and it is unclear if disruption in a network directly relates to decrease physiological output. It is entirely possible that examination of these gene networks by mRNA methods alone without better understanding of protein synthesis, glial cell function, and mechanisms is providing an inaccurate picture of myelin synthesis in these illnesses. As similar gene expression changes have been reported in bipolar disorder [37], it would be useful to examine this in future studies to contrast both the SZ and MDD findings.

### Methodology

The differences in the two groups in age, and PMI, fixation, though significant, were very small. The significance comes from the very tight selection of cases within these groups (SZ group mean age was only 3.3 years older than MDD group, Table 2). The SZ group was also in formalin fixation for longer than the MDD group, though this had no effect on the results. The previous studies on these disorders and this anatomical structure from the Corsellis collection have shown that formalin-fixation period does not affect neuropathological markers using histological staining [29, 42, 46, 47]. The lack of differences between the control and SZ groups in any measurement and the lack of significant interaction between confounding factors and results also suggest that any SZ and MDD difference is real and not an artifact of group variability.

It has been found that the nodes of Ranvier and the internodal sections of the axon increase in length in proportion to the diameter of the axon. In the CNS axon diameter varies from 0.2 to 20 µm and the myelin sheath is interrupted every 1–2 mm by a node of Ranvier approximately 2 µm in length. Our sections of 10, 100 µm apart, would not be affected by the size and distribution of the nodes. Increasing numbers of measurements of either myelin width or internal diameter past the optimum did not enhance the accuracy of the results. By measuring on two slides 100 µm apart, we negate any interacting effect of nodes.

When quantifying the width of myelin of irregular axons, the most precise data were obtained by measuring the width perpendicular to the intersection points. The lines provided by the asterisk were too long in many cases as they traversed the sheath at very shallow angles and, therefore, did not reflect the sheath width accurately. The use of a grid mask facilitated the unbiased selection of fixed points at which the width measurements were obtained, even when looking at irregular axons [29].

#### Limitations

Given the inherent difficulties in post-mortem studies, and the subsequent heterogeneity of results seen in many brain regions in neuropsychiatric studies, this investigation certainly should be repeated by independent investigators using brain tissue sourced from a different bank.

First, we have the issue of case selection, so commonly a difficult issue in neuropathology. Whilst the cases were extensively reviewed for inclusion by modern ICD-10 criteria with defined psychotic symptomatology and tightly age-matched, there was a significant sex bias between the groups. Although, statistically, this had no effect, ideally, this should be repeated with a greater balance of sexes to rule out any masked confounders.

Second, we used two methods to examine the amount of myelin at the level of measurement, thickness, and CSA. It could be reasonably said that these are two ways of measuring the same thing. However, the significant effect was only observed in CSA. This is likely to be due to the 2D effect measured by CSA being larger than the 1D effect measured by a simple line measure, rather than any actual significant biological difference. We anticipate that the reader will be able to make this distinction when drawing their conclusions from these results though.

### Future work

A repeat investigation of splenium axons is necessary as changes in FA have been noted in this structure [16]. It may be of interest to study the unmyelinated axons of the CC using antibodies against neurofilament to carry out immunofluorescence on these structures. The relative numbers of both types of axon in SZ, MDD, and control patients may also be significant as, perhaps, loss of one type is more pronounced than the other.
